# MicroRNA-199a-3p regulates proliferation and milk fat synthesis of ovine mammary epithelial cells by targeting *VLDLR*

**DOI:** 10.3389/fvets.2022.948873

**Published:** 2022-08-05

**Authors:** Jiqing Wang, Zhiyun Hao, Liyan Hu, Lirong Qiao, Yuzhu Luo, Jiang Hu, Xiu Liu, Shaobin Li, Fangfang Zhao, Jiyuan Shen, Mingna Li, Zhidong Zhao

**Affiliations:** Gansu Key Laboratory of Herbivorous Animal Biotechnology, College of Animal Science and Technology, Gansu Agricultural University, Lanzhou, China

**Keywords:** microRNA-199a-3p, VLDLR, ovine mammary epithelial cells, proliferation, triglyceride

## Abstract

In our previous study, microRNA (miR)-199a-3p was found to be the most upregulated miRNA in mammary gland tissue during the non-lactation period compared with the peak-lactation period. However, there have been no reports describing the function of miR-199a-3p in ovine mammary epithelial cells (OMECs) and the biological mechanisms by which the miRNA affects cell proliferation and milk fat synthesis in sheep. In this study, the effect of miR-199a-3p on viability, proliferation, and milk fat synthesis of OMECs was investigated, and the target relationship of the miRNA with very low-density lipoprotein receptor (*VLDLR*) was also verified. Transfection with a miR-199a-3p mimic increased the viability of OMECs and the number of Edu-labeled positive OMECs. In contrast, a miR-199-3p inhibitor had the opposite effect with the miR-199a-3p mimic. The expression levels of three marker genes were also regulated by both the miR-199a-3p mimic and miR-199-3p inhibitor in OMECs. Together, these results suggest that miR-199a-3p promotes the viability and proliferation of OMECs. A dual luciferase assay confirmed that miR-199a-3p can target *VLDLR* by binding to the 3′-untranslated regions (3'UTR) of the gene. Further studies found a negative correlation in the expression of miR-199a-3p with *VLDLR*. The miR-199a-3p mimic decreased the content of triglycerides, as well as the expression levels of six milk fat synthesis marker genes in OMECs, namely, lipoprotein lipase gene (*LPL*), acetyl-CoA carboxylase alpha gene (*ACACA*), fatty acid binding protein 3 gene (*FABP3*), *CD36*, stearoyl-CoA desaturase gene (*SCD*), and fatty acid synthase gene (*FASN*). The inhibition of miR-199a-3p increased the level of triglycerides and the expression of *LPL, ACACA, FABP3, SCD*, and *FASN* in OMECs. These findings suggest that miR-199a-3p inhibited milk fat synthesis of OMECs. This is the first study to reveal the molecular mechanisms by which miR-199a-3p regulates the proliferation and milk fat synthesis of OMECs in sheep.

## Introduction

The mammary gland is a special exocrine gland in mammals and is of economic importance. Mammary epithelial cells (MECs) around alveoli absorb nutrients from the blood and then convert them into milk. It is well-known that the viability, proliferation number, and secretory ability of MECs are responsible for milk yield and milk components, which in turn affect the survival rate, development, and pre-weaning growth rate of lambs, especially for non-dairy, multiple-born-lambs sheep breeds, such as Small-tailed Han sheep and Hu sheep farmed in China. The two breeds have average lambing rates of more than 250% per ewe. In this context, an in-depth knowledge of the molecular mechanisms that regulate MECs activities offers an opportunity to improve milk yield and quality, eventually resulting in an increase in the commercial return to sheep farmers.

It was found that the activity of MECs and mammary gland development are regulated by many functional genes and non-coding RNAs, including microRNAs (miRNAs). The length of miRNAs ranges from 18 to 25 nucleotides. They can complementally bind to the 3′-untranslated regions (3′UTR) of the target mRNAs to either induce degradation or inhibit translation, with an accompanying decrease of the target genes in expression (1). Increasing studies showed crucial roles of miRNAs in proliferation, apoptosis, and survival of MECs, as well as milk fat and milk protein synthesis in MECs (2). For example, miR-143 increased the content of triglycerides by targeting SMAD family 3 (*SMAD3*) (3). The miR-204-5p and miR-211 were found to coordinately inhibit the content of α-S1 casein (CSN1S1) (4). The effect of other miRNAs on MECs has also been reported, including miR-193a-5p (5), miR-128 (6), miR-106b (7), miR-221 (8), miR-16a (9), and miR-26a/b (10). However, these studies have been focused on dairy cows and dairy goats.

In sheep, the expression profiles of miRNAs in ovine mammary gland collected from different development periods or different breeds have been widely reported (11, 12), and some differentially expressed miRNAs were then screened. Although the functions of the differentially expressed miRNAs were investigated by enrichment analysis of their target genes, they have not been verified by experimental assays. At present, to the best of our knowledge, there are very few studies on the role of single miRNA in ovine mammary epithelial cells (OMECs) or mammary gland development in sheep. Hao et al. (13) found an inhibited effect of miR-432 on milk fat synthesis by targeting lipoprotein lipase (*LPL*) and stearoyl-CoA desaturase (*SCD*) genes. Nonetheless, it should be legitimately suggested that miRNAs are worthy of further investigation for a better understanding of molecular mechanisms underlying mammary gland development in sheep.

In our previous study, miR-199a-3p was found to be the most upregulated miRNA in the mammary gland during the non-lactation period when compared with the mammary gland at peak-lactation period, with the expression levels being 3.81-fold higher during the non-lactation (12). However, there have been no reports on the molecular mechanism of miR-199a-3p regulating mammary gland development and milk fat synthesis in sheep. Accordingly in this study, we investigated the effect of miR-199a-3p on viability, proliferation, and milk fat synthesis of OMECs, as well as the expression levels of some marker genes. We also confirmed the target binding of miR-199a-3p with very low-density lipoprotein receptor (*VLDLR*). These results will uncover the regulatory mechanism of miR-199a-3p underlying mammary gland development and milk fat synthesis in sheep.

## Materials and methods

### Animals and cell culture

Sample collection and cell treatment involved in this study were approved by the Animal Experiment Ethics Committee of Gansu Agricultural University, Lanzhou, China (Approval number GSAU-ETH-AST-2021-027). A healthy 3-year-old Small-tailed Han ewe was slaughtered at peak lactation (21 days postpartum), and mammary gland parenchyma samples were then collected.

The OMECs were isolated according to the method of Hao et al. (13). Briefly, cells were cultured in a basal DMEM/F12 medium (Invitrogen, CA, USA) consisting of 10% fetal bovine serum, 5 μg/ml hydrocortisone, 5 μl/ml insulin-transferrin-sodium selenium, 10 ng/ml epidermal growth factor 1, and 1 μg/ml hydrocortisone. To induce lactation of OMECs, they were then maintained in a lactogenic medium with the basal DMEM/F12 medium described above supplemented with 2 μg/ml prolactin (USBiological, MA, USA) for 48 h.

### Cell transfection, CCK8, and edu assays

A miR-199a-3p mimic, a miR-199a-3p inhibitor, and their negative controls (miR-199a-3p mimic NC and miR-199a-3p inhibitor NC) were synthesized by RiboBio Co. Ltd. (Guangzhou, China). When the confluence of OMECs reached ~80%, either the miR-199a-3p mimic (50 nM) or the miR-199a-3p inhibitor (100 nM) or their negative controls were transfected into the cells using INVI DNA & RNA Transfection Reagent™ (Invigentech, CA, USA). To investigate the effect of miR-199a-3p on the viability of OMECs, a 30 μl aliquot of CCK8 (Vazyme, Nanjing, China) was added to each well 46 h after transfection and then continued to incubate for 2 h. A Thermo Scientific Microplate Reader (Thermo Scientific, MA, USA) was used to measure the absorbance of OMECs at 450 nm. Four wells were selected from each group of cells treated to count their cell viability.

To detect the proliferation of OMECs regulated by miR-199a-3p, an Edu assay was performed 44 h after transfection with the miR-199a-3p mimic and miR-199a-3p inhibitor. Briefly, 100 ml of 50 mM Cell-Light™ Edu reagent (Beyotime, Shanghai, China) was added to each well and then cultured for 4 h. A microscope IX73 (Olympus, Tokyo, Japan) was used to observe the Edu staining result, and the percentage of Edu-labeled positive OMECs was calculated.

### RT-qPCR

To investigate the transfection efficiency of miR-199a-3p, as well as its roles in proliferation and milk fat synthesis of OMECs, reverse transcription-quantitative PCR (RT-qPCR) analysis was performed. Briefly, when OMECs grew to 70–80%, the cells were transfected with the miR-199a-3p mimic, miR-199a-3p inhibitor, miR-199a-3p mimic NC, and miR-199a-3p inhibitor NC. Total RNA was isolated from transfected OMECs using TRIzol reagent (Invitrogen, CA, USA), and then reverse transcribed to produce cDNA using SuperScriptTM II reverse transcriptase kit (Invitrogen, CA, USA). The RT-qPCR was carried out in triplicate using an SYBR qPCR Master Mix (Vazyme, Nanjing, China). *GAPDH* was chosen as an internal reference for standardization as suggested by Jiao et al. (8). A 2^−ΔΔCt^ method was used to calculate the relative expression levels of miR-199a-3p and 10 genes, namely, *VLDLR*, BCL2 apoptosis regulator (*BCL-2*), BCL2-associated X (*Bax*), *Caspase3, LPL*, acetyl-CoA carboxylase alpha (*ACACA*), fatty acid binding protein 3 (*FABP3*), *CD36, SCD*, and fatty acid synthase (*FASN*). Their PCR primers are listed in Table 1.

**Table 1:** PCR primers used in the study.

**Name**	**Forward (5^′^ → 3^′^)**	**Reverse (5^′^ → 3^′^)**	**Amplicon size (bp)**
*VLDLR* ^a^	CCGCTCGAGATTCCCATCCACATTCTC	GAATGCGGCCGCGCCATATTATAGGCTTCATT	573
*VLDLR* ^b^	GTTCAATGATGACTACATTTTTTTTCCAAGTGCTAAAAAA	TGTAGTCATCATTGAACACTTAGTCTTTGCAAACCTC	573
*VLDLR^*c*^*	CGTTGTCTGGCTTTGTTT	GGTCAAGTGTAATTCCGTTAG	131
*Bax*	GAGATGAATTGGACAGTAACATGGA	CAAAGTAGAAAAGGGCGACAAC	150
*Caspase3*	GGCTCTGAGTGTTTGGGGAA	CCTGGACAAAGTTCCGTGGT	131
*Bcl-2*	GGATGACCGAGTACCTGAACC	CATACAGCTCCACAAAGGCATC	80
*SCD*	AGATTTATCCGACCTAAGAGC	CCATAGATACCACGGCAC	115
*FABP3*	TCACTCGGTGTCGGTTTT	CATCTGCCGTGGTCTCAT	166
*CD36*	TGGATTTACTTTACGGTTTG	GCCCAGGAGGTTTATTTT	196
*ACACA*	GTCCTCTGCCAGTTTCCC	TCCATCACCACAGCCTTC	173
*LPL*	ACCTGAAGACTCGTTCTC	CACCTCCGTGTAAAGTAG	207
*FASN*	GCGTTCCACTCCTATTTC	CACCAGGTTGTTCACATT	180
*GAPDH*	ATCTCGCTCCTGGAAGATG	TCGGAGTGAACGGATTCG	227
*miR-199a-3p*	ACAGTAGTCTGCACATTGGTT	mRQ 3′ primer^d^	/

^a^*The primers were used to amplify the 3′-untranslated regions (3′-UTR) of VLDLR when a wild-type pmirGLO vector was constructed*.

*^b^The primers were used to amplify mutated sequences of the 3′-UTR of VLDLR bound with miR-199a-3p when a mutant pmirGLO vector was constructed*.

*^c^The primers designed for RT-qPCR*.

*^d^A universal reverse primer used for Poly(T) adaptor RT-qPCR analysis of a miRNA*.

### Dual luciferase reporter assay

The *VLDLR* was predicted to be a target gene of miR-199a-3p using the Targetscan 3.1 and miRanda 3.3a. To verify the target interaction of miR-199a-3p with *VLDLR*, a dual luciferase reporter assay was performed. Briefly, a 573-bp fragment in the 3′UTR of *VLDLR* containing the target site of miR-199a-3p predicted was amplified from OMECs cDNA using PCR primers (Table 1). The amplified product was inserted into the SacI and Xhol restriction sites of a pmirGLO vector to produce a wild-type pmirGLO dual luciferase reporter vector. Among the 573-bp 3′UTR of *VLDLR*, the target binding sequences for miR-199a-3p were complementarily mutated from “ACTACTG” to “TGATGAC,” thereby producing a mutant pmirGLO dual luciferase reporter vector. The two vectors were validated by electrophoresis in 1.5% agarose gels and Sanger sequencing.

A total of 0.20 μg plasmid was extracted from the wild-type or mutant pmirGLO dual luciferase reporter vector, and 20 pmol of miR-199a-3p mimic or miR-199a-3p mimic NC were co-transfected into the HEK293 T cells using INVI DNA & RNA Transfection Reagent™ (Invigentech, CA, USA). After transfection for 48 h, the Renilla and firefly luciferase activities of *VLDLR* were detected using the Dual-Luciferase Reporter Kit (Promega, WI, USA) by a Varioskan LUX Microplate Reader (Thermo Lifetech, MA, USA).

### Triglyceride content analysis

For the OMECs transfected with the miR-199a-3p mimic, miR-199a-3p inhibitor, miR-199a-3p mimic NC, and miR-19a-3p inhibitor NC, the supernatant was collected by centrifugation from 300 μl of lysate solution added 48 h after transfection. The content of triglycerides in OMECs was measured in quadruplicate using a triglyceride content assay kit (Solarbio, Beijing, China) by a microplate reader (Thermo Lifetech, MA, USA).

### Statistical analysis

The difference between the two groups was compared using two-tailed Student's *t*-test in SPSS 22.0 (IBM, NY, USA). Data are indicated as mean ± standard error of the mean (SEM). All *P*-values were considered statistically significant when *P* < 0.05.

## Results

### miR-199a-3p increases viability of OMECs

**Figure 1:**
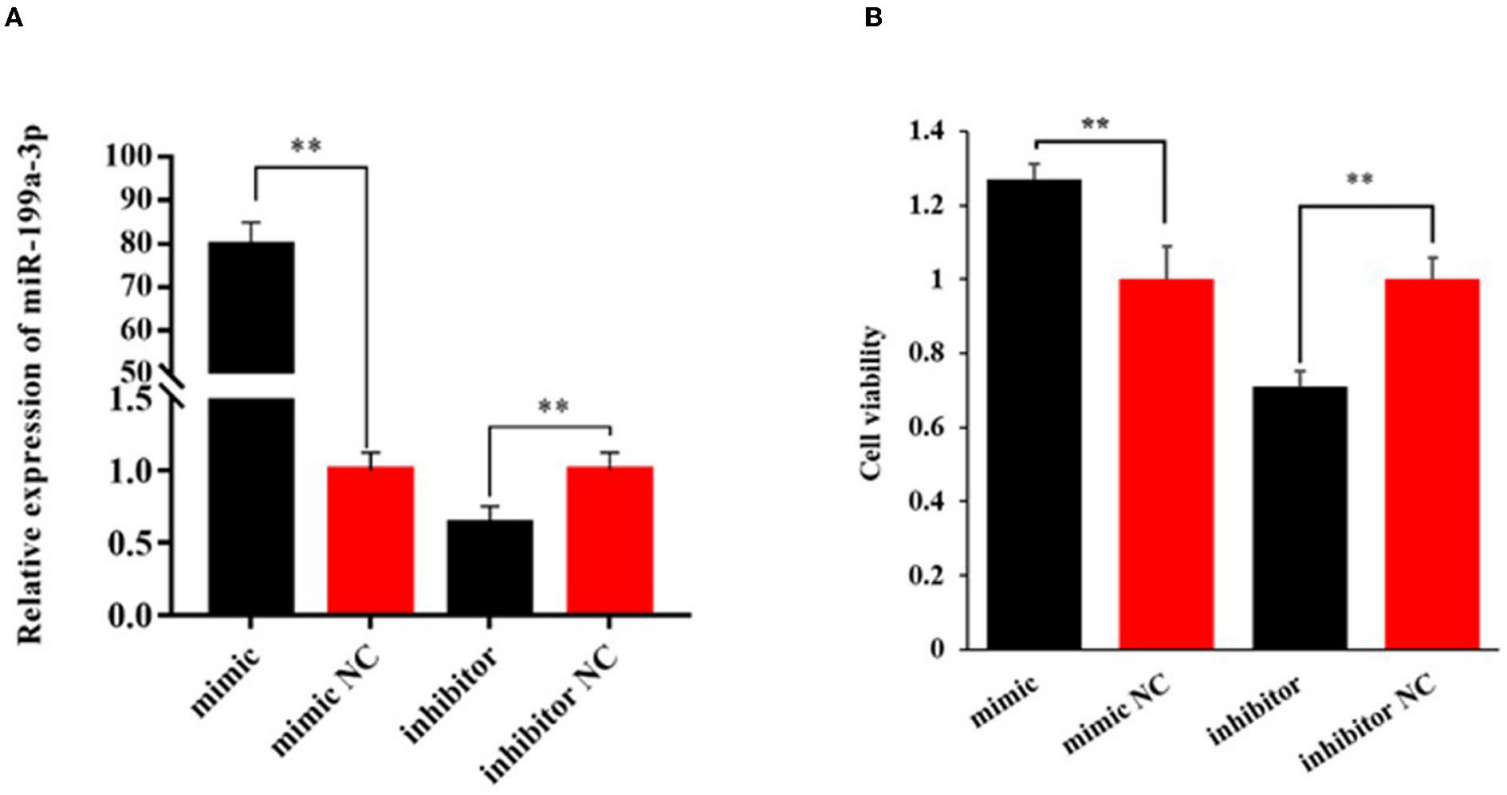
Relative expression level of miR-199a-3p **(A)** and its effect on the viability of ovine mammary epithelial cells (OMECs) **(B)** when miR-199a-3p mimic and miR-199a-3p inhibitor were transfected into OMECs. **P < 0.01.

To explore the effect of miR-199a-3p on viability, proliferation, and milk fat synthesis of OMECs, the miR-199a-3p mimic, miR-199a-3p inhibitor, and their NC were transfected into OMECs. The RT-qPCR analysis revealed that the expression of miR-199a-3p was 80-fold higher in the miR-199a-3p mimic group compared with miR-199a-3p mimic NC. In contrast, compared with miR-199a-3p inhibitor NC, miR-199a-3p had a significant decrease in the expression of OMECs transfected with miR-199a-3p inhibitor (Figure 1A). The results suggest that miR-199a-3p mimic and miR-199a-3p inhibitor were successfully transfected into OMECs.

The CCK8 assay found that cell viability significantly increased in OMECs transfected with miR-199a-3p mimic when compared with miR-199a-3p mimic NC, while miR-199a-3p inhibitor resulted in a significant decrease in cell viability compared with its NC group (*P* < 0.01, Figure 1B).

### miR-199a-3p promotes proliferation of OMECs

**Figure 2:**
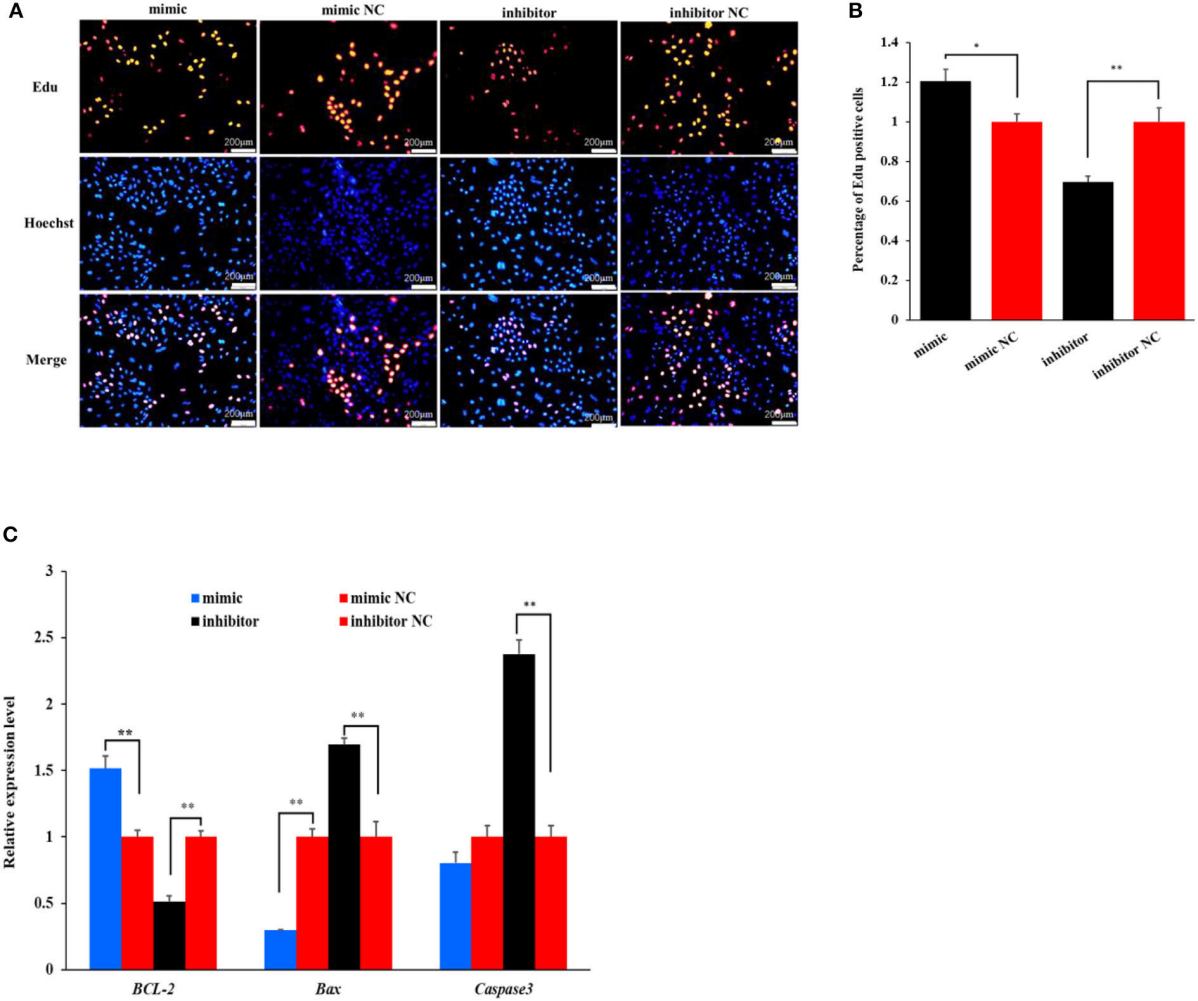
The effect of miR-199a-3p on the proliferation of ovine mammary epithelial cells (OMECs) when miR-199a-3p mimic and miR-199a-3p inhibitor were transfected into OMECs. **(A)** The proliferation of OMECs detected using an Edu assay. **(B)** The percentage of Edu-labeled positive OMECs counted by the ImageJ software. **(C)** The expression levels of *BCL-2, Bax*, and *Caspase 3*. **P < 0.01 and *P < 0.05.

The Edu analysis revealed that the percentage of Edu-labeled positive OMECs was increased by 20.43% in the miR-199a-3p mimic group compared with its NC group (*P* < 0.05), while the percentage was decreased by 30.29% in the miR-199a-3p inhibitor group (*P* < 0.01, Figures 2A,B).

To further validate the effect of miR-199a-3p on cell proliferation, the expression levels of three marker genes were detected using RT-qPCR. As shown in Figure 2C, miR-199a-3p mimic increased the expression of *BCL-2*, while the inhibition of miR-199a-3p had the opposite effect on the gene in expression (*P* < 0.05). Meanwhile, miR-199a-3p mimic inhibited the expression of *Bax* (*P* < 0.05), and 199a-3p inhibitor led to an increase in the expression level of *Caspase3* (*P* < 0.05, Figure 2C). Taken together, these results suggest that miR-199a-3p promotes the proliferation of OMECs in sheep.

### miR-199a-3p targets the 3′UTR of VLDLR

**Figure 3:**
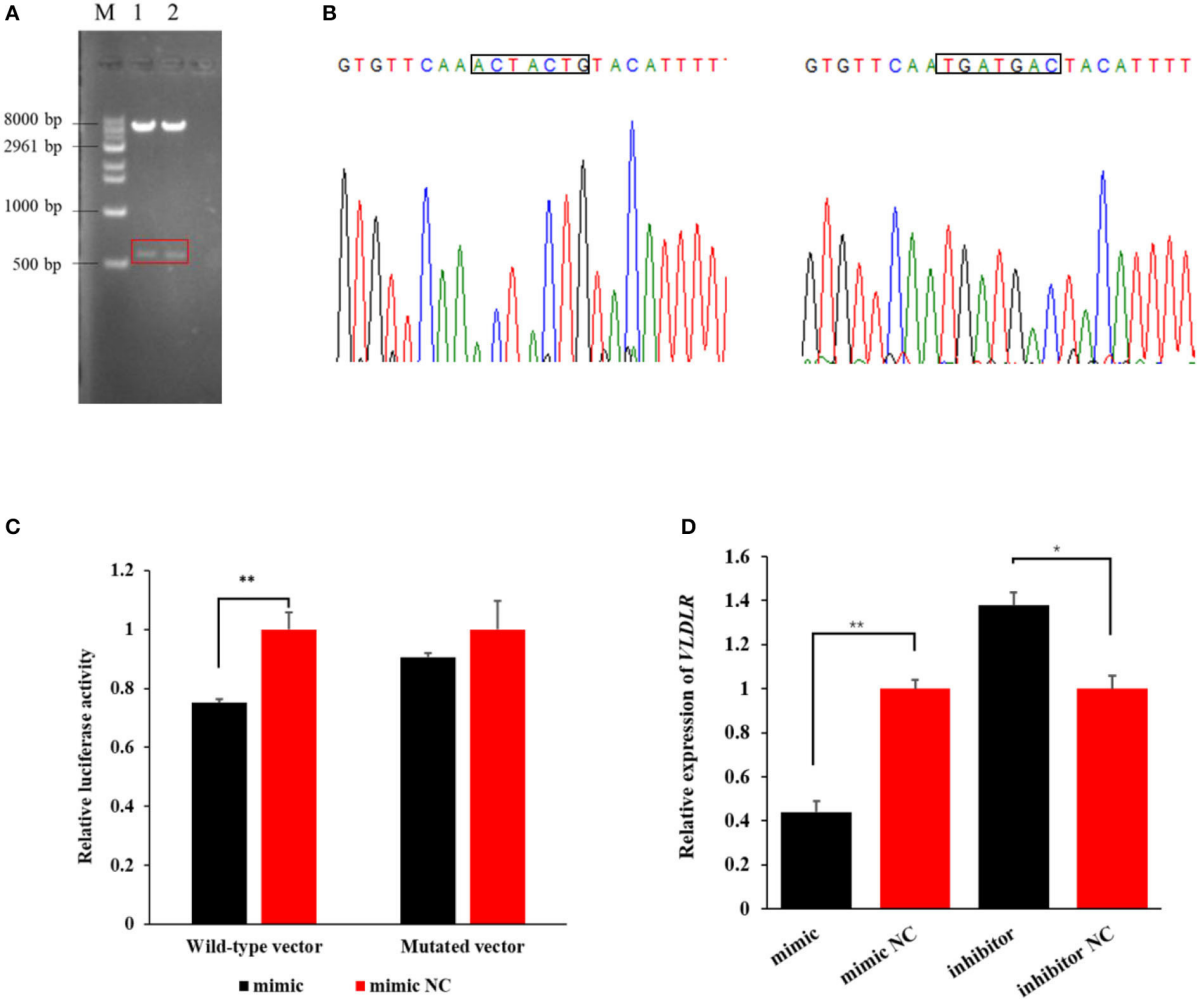
The target relationship verification of miR-199a-3p with *VLDLR* using a dual-luciferase reporter assay. **(A)** Dual luciferase reporter vectors after restriction endonucleases digestion were visualized using agarose gel electrophoresis. M: marker; 1: wild-type vector; 2: mutant vector. **(B)** The Sanger sequencing result of sequences of wild-type and mutant vectors. **(C)** The luciferase activity of *VLDLR* was detected when the miR-199a-3p mimic or miR-199a-3p mimic NC, and wild-type or mutant dual luciferase reporter vectors were co-transfected into HEK293T cells. **(D)** Effect of miR-199a-3p mimic and miR-199a-3p inhibitor on the expression level of *VLDLR* in ovine mammary epithelial cells (OMECs). **P < 0.01 and *P < 0.05.

The agarose gel electrophoresis result revealed that amplicons of the expected size (~573 bp) were successfully ligated into dual luciferase reporter vectors (Figure 3A). The result from Sanger sequencing further confirmed the presence of the binding sequence in the 3′UTR of *VLDLR* with miR-199a-3p in the wild-type pmirGLO vector, as well as mutated sequences in the mutant vector (Figure 3B). This suggests that the two vectors were constructed as expected.

In the group of co-transfections with wild-type pmirGLO vector and miR-199a-3p mimic, the luciferase activity of *VLDLR* significantly decreased to 75% of the negative control group (*P* < 0.01). However, in the HEK293 T cells transfected with the mutant pmirGLO vector, there was no significant difference in the luciferase activity of *VLDLR* between miR-199a-3p mimic and miR-199a-3p mimic NC (*P* > 0.05, Figure 3C). This suggests that miR-199a-3p targets *VLDLR* by binding to its 3′UTR.

The RT-qPCR analysis revealed that miR-199a-3p mimic decreased the expression of *VLDLR* (*P* < 0.01), while the inhibition of miR-199a-3p led to the upregulation of *VLDLR* expression in OMECs (*P* < 0.05, Figure 3D).

### miR-199a-3p inhibits milk fat synthesis

To investigate the effect of miR-199a-3p on milk fat synthesis, both the content of triglycerides and the expression levels of six milk fat synthesis marker genes, namely, *LPL, ACACA, FABP3, CD36, SCD*, and *FASN*, were detected in OMECs transfected with miR-199a-3p mimic and miR-199a-3p inhibitor.

**Figure 4:**
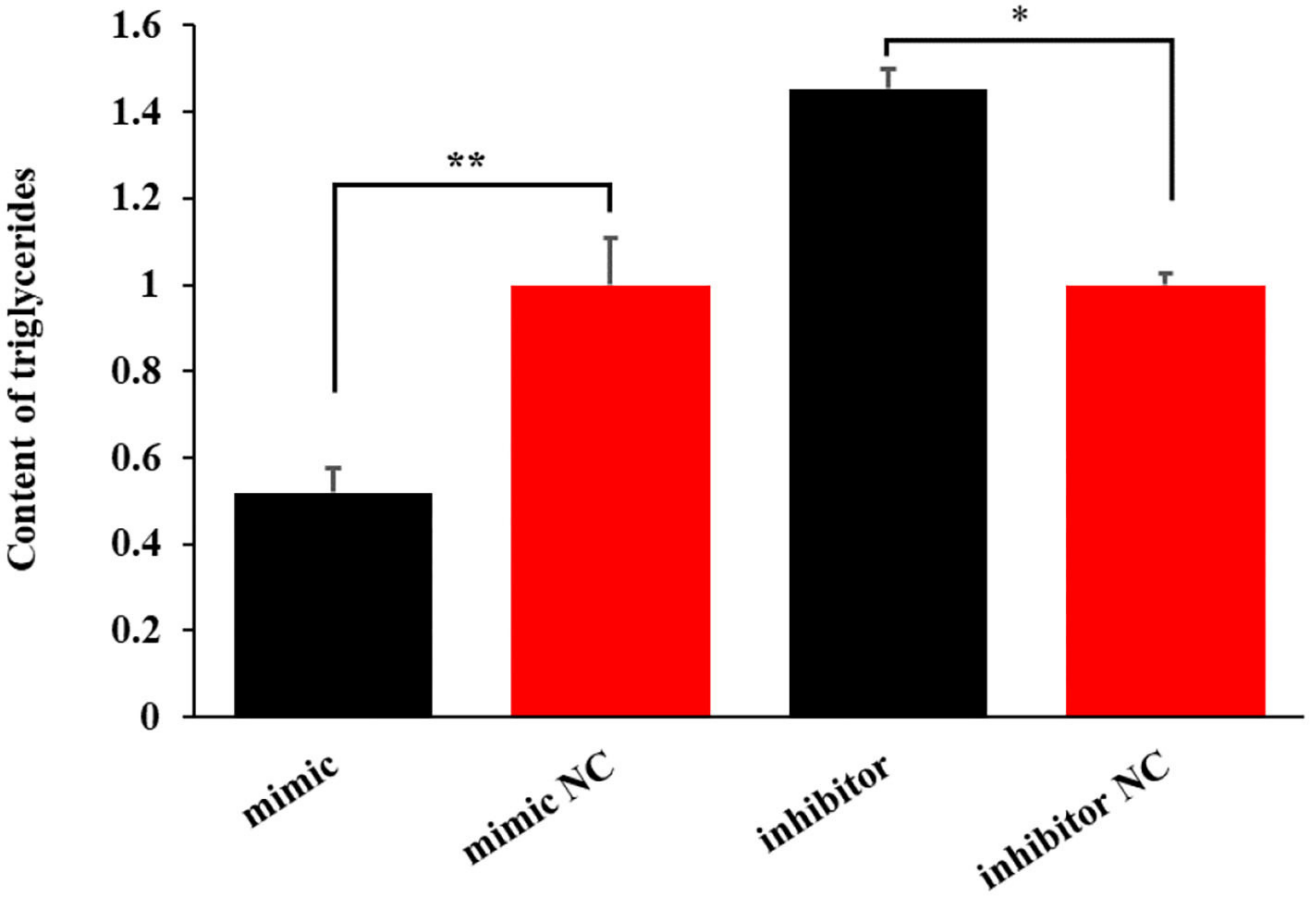
The effect of miR-199a-3p on the content of triglycerides in ovine mammary epithelial cells (OMECs). **P < 0.01 and *P < 0.05.

The miR-199a-3p mimic decreased the level of triglycerides by 48.02% (*P* < 0.01). In contrast, the inhibition of the miRNA increased the content of triglycerides by 45.27% in OMECs (*P* < 0.05, Figure 4).

**Figure 5:**
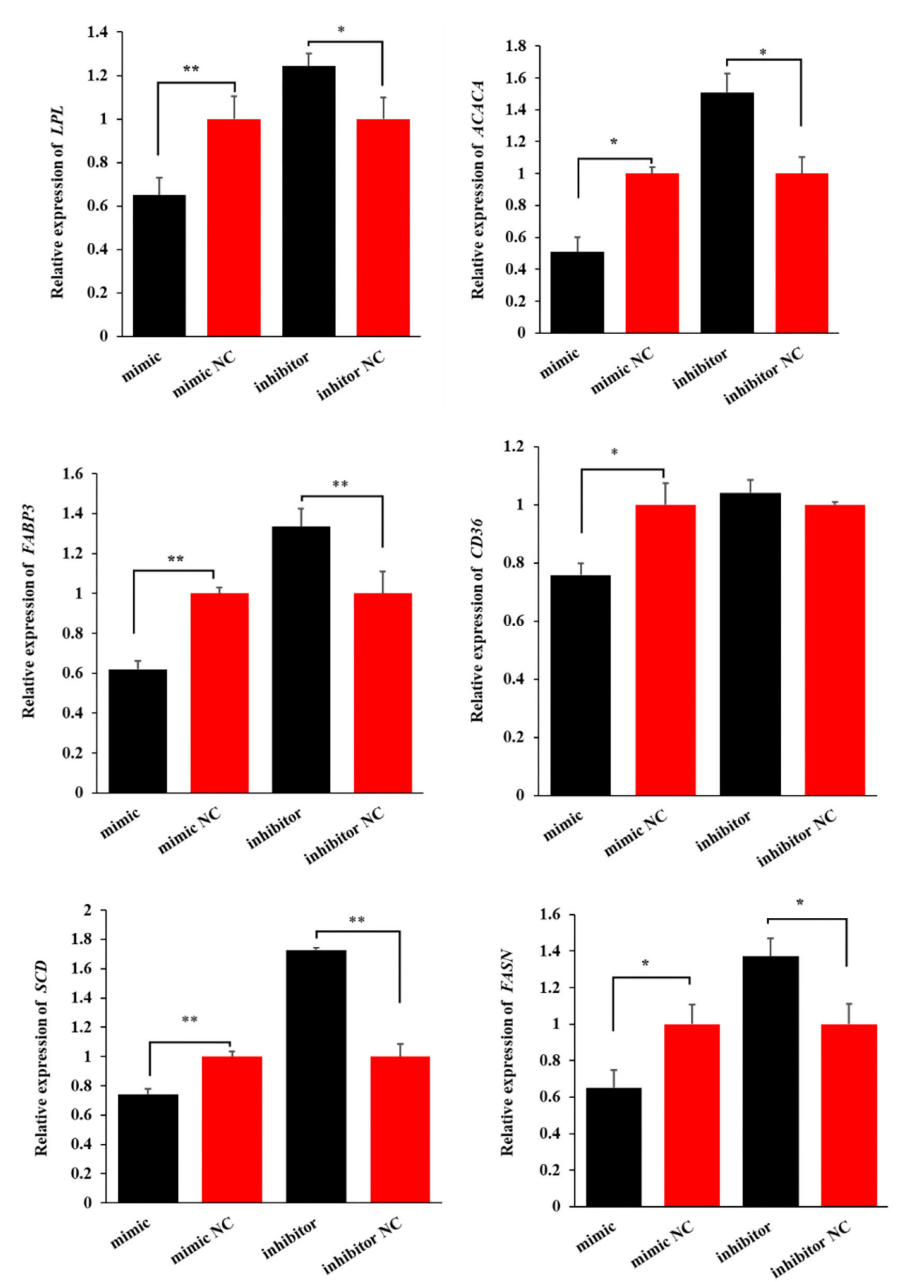
The expression levels of six milk fat synthesis marker genes, namely, *LPL, ACACA, FABP3, CD36, SCD*, and *FASN*, when the miR-199a-3p mimic and miR-199a-3p inhibitor were transfected into ovine mammary epithelial cells (OMECs). **P < 0.01 and *P < 0.05.

Similarly, miR-199a-3p mimic decreased the expression levels of the six genes, while miR-199a-3p inhibitor increased the expression of *LPL, ACACA, FABP3, SCD*, and *FASN* in OMECs (Figure 5). Taken together, these results suggest that miR-199a-3p inhibits milk fat synthesis of OMECs in sheep.

## Discussion

This study investigates the effect of miR-199a-3p on the proliferation and milk fat synthesis of OMECs, as well as its regulatory mechanism underlying the cells. At present, research into the effect of miR-199a-3p on cell proliferation has primarily been focused on cancer cells and stem cells in humans, but the findings obtained were controversial. Most studies found that miR-199a-3p inhibited the proliferation of hepatocellular carcinoma, colorectal cancer, and prostate cancer cells (14). An inhibition role of miR-199a-3p in the proliferation of human embryonic stem cells has also been reported (15). However, there are exceptions. Shatseva et al. (16) found that miR-199a-3p expression increased the viability and proliferation of both breast cancer cells and endothelial cells. The promoted effect of miR-199a-3p on cell proliferation has also been reported in gastric cancer cells (17) and adipocytes of mice (18). The last findings supported our results about the promotion of miR-199a-3p in cell viability and proliferation of OMECs. It has been reported that the proliferation number and viability of mammary epithelial cells are positively related to their milk yield in mammals (11). In this context, it was speculated that miR-199a-3p may improve milk yield in sheep. The contention is supported by the observation that miR-199a-3p was found to be upregulated in Kazakh horse with higher milk yield (19).

It was notable that miR-199a-3p regulated the expression of *BCL-2, Bax*, and *Caspase 3* in OMECs. These three genes are related to cell survival or apoptosis. BCL-2 is an anti-apoptotic protein and is able to inhibit apoptosis of MECs induced by a variety of stimuli. In a gain-of-function transgenic mouse model, *BCL-2* gain of function significantly decreased the number of apoptotic MECs but increased the survival rate of MECs during mammary gland involution periods (20). Meanwhile, over-expressed *BCL-2* was found to increase the number of MECs in transgenic mice (21). In this respect, the result that miR-199a-3p enhanced the expression of *BCL-2* further supported the promoted effect of miR-199a-3p on the proliferation of OMECs in the study. Bax and Caspase 3 are pro-apoptotic effector members. Over-expressed *Bax* has been demonstrated to induce apoptosis of MECs (22). Caspase 3, an endoprotease, is a crucial regulator of programmed cell death. The knockout of *Caspase 3* led to increases in proliferation *in vivo* and hyperproliferation after mitogenic stimulation *in vitro* (23). These suggest a negative correlation between the expression of *Caspase 3* with cell proliferation. Upon our joint analysis of the Targetscan 3.1 and miRanda 3.3a, no target relationships between miR-199a-3p and the three marker genes were predicted. Additionally, although miR-199a-3p can target *VLDLR*, the protein VLDLR mainly plays a biological role in fat synthesis. It has not been reported that VLDLR or the pathway *via* VLDLR regulated cell proliferation or viability. It is, therefore, impossible that miR-199a-3p regulates the proliferation and viability of OMECs by targeting *VLDLR*. Taken together, it was, therefore, inferred that miR-199a-3p promoted the proliferation and viability of OMECs by regulating the expression of *BCL-2, Bax*, and *Caspase 3*. However, the molecular mechanism underlying the process would need to be investigated.

Triglycerides are the main component of milk fat globules located in mammary epithelial cells, eventually forming milk fat. In this study, miR-199a-3p decreased the content of triglycerides in OMECs. The result was in accordance with the observation that over-expressed miR-199a-3p had the capability to weaken lipid accumulation and adipogenic gene expression in preadipocytes (24) and 3T3-L1 adipocytes (18) in mice. The miRNA was found to be one of the upregulated miRNAs identified in the bovine mammary gland, for Holstein dairy cows fed diets supplemented with either linseed or safflower oil that significantly decreased milk fat (25). The miR-199a-3p has been reported to exhibit a higher expression level during the non-lactating period than at lactation in ewes (12) and dairy cows (26). Given that more fat droplets originated from triglycerides at lactation when compared with the non-lactation period, these were also consistent with our observation. However, it was reported that over-expressed miR-199a-3p accelerated adipogenesis of bone narrow-derived mesenchymal stem cells (27). The inconsistent results, therefore, suggest that the effect of miR-199a-3p on lipogenesis should be further investigated in different kinds of cells.

This is the first study to report the target binding of miR-199a-3p with *VLDLR*. *VLDLR* was found to be expressed in the bovine mammary gland (28), which was in agreement with our results. The protein VLDLR is a member of the low-density lipoprotein receptor family. VLDLR can bind apolipoprotein E (apoE) triglyceride-rich lipoproteins and then accelerate subsequent triglyceride hydrolysis by LPL, resulting in the entry of lipid into MECs (29). Meanwhile, VLDLR also strengthens the activity of LPL that releases long-chain fatty acids from chylomicron and very low-density lipoprotein. Long-chain fatty acids are used as raw materials for synthesizing triglycerides in MECs (13). In addition, knockout mice of *VLDLR* exhibited a decrease in adipocyte triglyceride storage (30). Taken together, these studies suggest that *VLDLR* may be responsible for triglyceride synthesis. In this study, it was observed that over-expression of miR-199a-3p decreased *VLDLR* expression in OMECs. These results confirmed again the inhibited effect of miR-199a-3p on triglyceride content of OMECs by targeting *VLDLR*.

Besides *VLDLR*, miR-199a-3p reduced the expression levels of *FASN, ACACA, CD36, LPL, FABP3*, and *SCD* of OMECs in this study. These genes were all associated with milk fat synthesis. Milk fat synthesis in OMECs consisted of exogenous fatty acid absorption, *de novo* synthesis of fatty acid, and synthesis and secretion of triglycerides. The rate-limiting enzymes, FASN and ACACA, are involved in *de novo* lipogenesis in OMECs. CD36 is a fatty acid transferase, and it contributes to the absorption of long-chain fatty acids in OMECs when entering the extracellular environment (31). Non-esterified fatty acids carried by chylomicrons, very low-density lipoprotein, and albumin are hydrolyzed into long-chain fatty acids by LPL (32), followed by intracellular transport for esterification by FABP3 (33). SCD is a key factor in the synthesis of monounsaturated fatty acids, which is further used for producing triglycerides in OMECs. It was, therefore, concluded that downregulation of *FASN, ACACA, CD36, LPL, FABP3*, and *SCD* in the expression by miR-199a-3p was responsible for the inhibition of milk fat synthesis in OMECs.

In addition to mammary gland tissues, miR-199a-3p was found to be expressed in hair follicles (16), cardiac myocytes (34), ovary (35), and adipose tissues (24). The miRNA has been verified to play biological functions in the regulation of hair follicles development, tumorigenesis, and adipocyte differentiation by targeting caveolin 2 (*CAV2*), zinc fingers and homeoboxes 1 (*ZHX1*), *SCD*, and mechanistic target of rapamycin kinase (*mTOR*) (16–18, 24). These studies affirm that a single miRNA appears to target a range of different mRNAs and suggest that miR-199a-3p may play pleiotropic roles in a variety of cellular activities and biological processes by targeting different functional genes.

## Conclusion

Our results suggest that miR-199a-3p promotes the viability and proliferation of OMECs, while it inhibits milk fat synthesis of OMECs by targeting *VLDLR*. This study provides a better understanding of the functions of miR-199a-3p in ovine mammary gland development and milk fat synthesis.

## Data availability statement

The original contributions presented in the study are publicly available. This data can be found here: NCBI [accession: ON572243 and ON572244].

## Ethics statement

The animal study was reviewed and approved by Animal Experiment Ethics Committee of Gansu Agricultural University, Lanzhou, China (Approval Number GSAU-ETH-AST-2021-027). Written informed consent was obtained from the owners for the participation of their animals in this study.

## Author contributions

JW contributed to the data analysis and wrote the manuscript. ZH, LH, LQ, YL, and JH performed the investigation and collected the samples. XL, SL, FZ, JS, ML, and ZZ performed the formal analysis, methodology, and software analysis. JW did the project administration and revised the manuscript. All authors contributed to the article and approved the submitted version.

## Funding

This study was financially supported by the National Natural Science Foundation of China (32060746 and 31860635), the Yong Supervisor Support Fund of Gansu Agricultural University (GAU-QDFC-2020-01), the Fuxi Young Talents Fund of Gansu Agricultural University (Gaufx-02Y02), and the Science and Technology Project of Lanzhou City (2021-1-162).

## Conflict of interest

The authors declare that the research was conducted in the absence of any commercial or financial relationships that could be construed as a potential conflict of interest.

## Publisher's note

All claims expressed in this article are solely those of the authors and do not necessarily represent those of their affiliated organizations, or those of the publisher, the editors and the reviewers. Any product that may be evaluated in this article, or claim that may be made by its manufacturer, is not guaranteed or endorsed by the publisher.
